# Shellfish Aquaculture Complements Nutrient Reduction in Mitigating Climate-Exacerbated Coastal Hypoxia

**DOI:** 10.1021/acs.est.5c06682

**Published:** 2025-08-21

**Authors:** Liuqian Yu, Jianping Gan, Dou Li, Weicong Cheng, Ying Zhang, Hiusuet Kung, Chiwing Hui, Zheng Chen

**Affiliations:** † Center for Ocean Research in Hong Kong and Macau, 58207 The Hong Kong University of Science and Technology, Kowloon, Hong Kong 999077, China; ‡ Earth, Ocean and Atmospheric Sciences Thrust, 567841 The Hong Kong University of Science and Technology (Guangzhou), Guangzhou 511453, China; § Department of Ocean Science and Department of Mathematics, 1846 The Hong Kong University of Science and Technology, Kowloon, Hong Kong 999077, China; ∥ Department of Earth and Environment, Boston University, Boston, Massachusetts 02215, United States

**Keywords:** coastal hazard, eutrophication, nutrient management, climate projection, water column stratification, ecosystem modeling

## Abstract

Coastal hypoxia, driven by human-induced nutrient enrichment and global warming, significantly threatens marine ecosystems. While cutting land-derived nutrient sources has been proposed as the key solution, its effectiveness may be undermined by climate change, and costs may rise after addressing the easier targets. Evaluating various nutrient measures under future climate scenarios is critical but challenging due to inadequate projections for coastal processes. In this global context, we combine field measurements with a coast-resolved physical-biogeochemical model to assess the effectiveness of land-based and alternative mitigation strategies under near-term (2016–2045) and long-term (2071–2100) climate change scenarios in a representative estuary-shelf system. Our findings evidence that stringent land-based nutrient reduction is necessary but insufficient to halt deoxygenation under climate change. This insufficiency is mainly due to enhanced water-column stratification, driven by climate warming and its associated increase in river discharge, which restricts oxygen replenishment more than the reductions in biogeochemical oxygen consumption achieved through nutrient management. We demonstrate that oyster aquaculture can serve as an effective complementary strategy to remove nutrients and combat oxygen depletion. This incentivizing approach aligns with the global trend of increasing nonfed aquaculture, offering a promising supplementary solution to the challenges of managing coastal nutrients and mitigating hypoxia globally.

## Graphical abstract

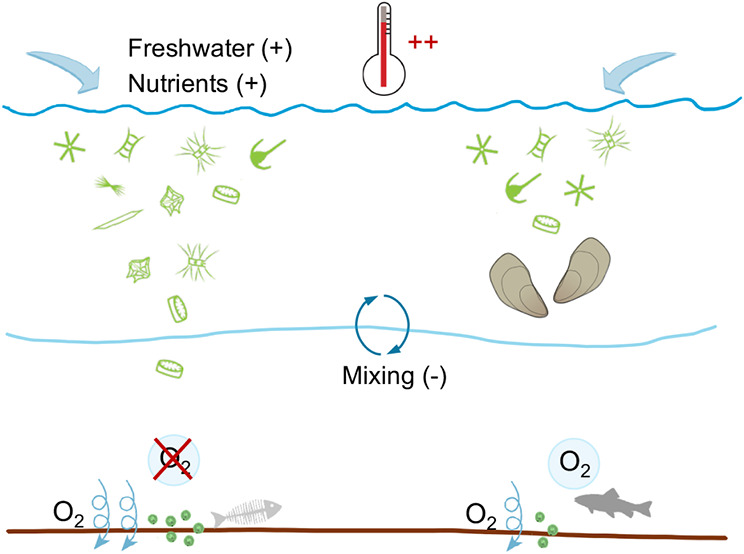

## 1 Introduction

Coastal oceans are rapidly losing oxygen (Figure 1). This deoxygenation disrupts global biogeochemical cycles, adversely affects marine life, and undermines essential ecosystem services for humans.^1,2^ The primary driver of this widespread phenomenon is the massive increase in anthropogenic nutrient discharge into coastal waters, which stimulates organic matter production and accelerates bottom-water oxygen consumption. As a result, the global expansion of coastal eutrophication and hypoxia (dissolved oxygen <2 mg/L) is perceived to correlate closely with the pace of socioeconomic development worldwide.^3,4^ For example, coastal hypoxia was sparsely reported in North America and northern Europe before the 1970s but became prevalent in the 1990s. Since the 2000s, coastal hypoxia has also plagued developing countries in South and East Asia, following the surge in synthetic fertilizer use, and is likely expanding to Africa and South America.^4,5^ Nevertheless, these emerging hypoxic zones are not as well-monitored as those in developed nations, contributing to the underreporting of hypoxic sites in developing regions despite their high nutrient loads (Figure 1A).

**Figure 1:**
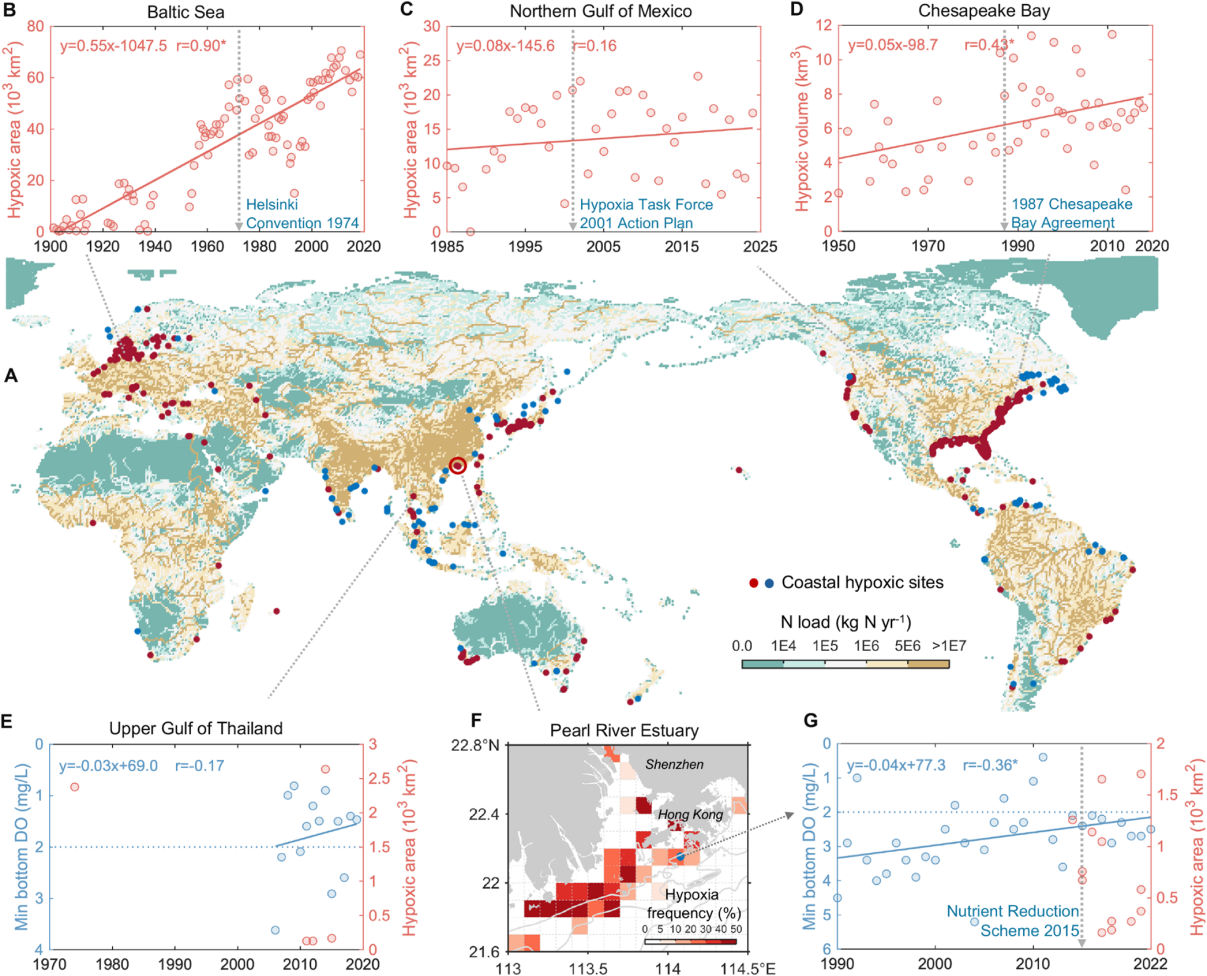
Global distribution of nitrogen delivery and coastal hypoxic sites, along with hypoxic conditions in select coastal zones. (A) Global distribution of nitrogen delivery to surface waters in 2020, modeled under the Shared Socio-economic Pathways (SSP) scenario SSP1,^21^ alongside locations of coastal hypoxic zones: 476 sites in red (from Diaz and Rosenberg^22^) and 117 sites in blue (synthesized from literature by this study). (B– E, and G) Temporal evolution and trends of hypoxic extent in (B) Baltic Sea,^23^ (C) Northern Gulf of Mexico,^24^ (D) Chesapeake Bay,^25^ (E) the Upper Gulf of Thailand,^4^ and (G) Pearl River Estuary (PRE). In panels (E) and (G), the annual bottom oxygen minimum is shown for time-series monitoring sites in the Upper Gulf of Thailand (100.59°E and 13.52°N) and PRE (blue dot in panel F), respectively. Solid lines represent the linear fit of observations, with asterisks (*) indicating correlation coefficients significant at *p* < 0.05. Vertical dashed arrows mark the year when large-scale nutrient management plans or agreements were launched in the respective watershed. (F) Frequency of hypoxia estimated from 13 summer surveys conducted from 2014 to 2021, covering the PRE and adjacent shelf.

Reducing land-based nutrient inputs has long been viewed as essential for alleviating eutrophication-driven hypoxia (see nutrient reduction schemes in Figure 1B,C,D,G). However, historical data indicate that coastal hypoxic systems rarely respond linearly to nutrient reductions.^6^ This nonlinear response is partially due to physical controls on hypoxia,^7,8^ governed by complex interactions among oceanic processes such as river plumes, tides, and wind-driven coastal currents, and climate stressors like warming and changes in precipitation and prevailing winds.^9^ These processes regulate oxygen ventilation by altering water column stratification and mixing while also affecting biogeochemical oxygen production and consumption by distributing nutrients and organic matter and modifying environmental conditions for phytoplankton growth and trophic interactions. Additionally, on longer time scales, climate change complicates the physical and biogeochemical response of coastal waters to nutrient inputs, as warming can decrease oxygen solubility, intensify water column stratification, alter circulation, and enhance oxygen consumption through microbial respiration.^1^ These changes may undermine the effectiveness of nutrient reduction strategies in combating coastal hypoxia.^10^

More stringent nutrient reduction strategies are thus essential for addressing the challenges posed by a warming future; however, their implementation encounters several socioeconomic and technical obstacles. First, developing regions often struggle to balance socioeconomic development with the need for environmental pollution control, which hampers strict regulatory enforcement. Second, in developed regions that have already made progress in reducing nutrient inputs, easier measures, such as cutting point-source emissions, have largely been exhausted. This leaves the more challenging and costly task of reducing diffuse emissions to further mitigate nutrient pollution. Finally, conventional land-based nutrient control measures cannot remove nutrients already discharged into aquatic systems or those released from sediments.^11^

Given these challenges, *in situ* mitigation measures are increasingly advocated to complement land-based source control approaches.^12^ Examples include nonfed aquaculture such as kelp,^13^ seaweed,^14^ and shellfish,^15,16^ along with oyster reef restoration,^17^ and artificial oxygenation.^18^ Among them, cultivating filter-feeding shellfish has been shown to reduce eutrophication and control phytoplankton blooms in various coastal systems.^19^ Additionally, the growing human demand for seafood protein creates strong incentives for shellfish cultivation,^20^ making it a promising win-win for both the economy and ecosystems. However, the effectiveness of large-scale shellfish aquaculture compared to land-based source control for mitigating hypoxia under future climate conditions is rarely quantified as global climate models cannot adequately capture the regional-scale estuarine processes critical for simulating coastal hypoxia and shellfish aquaculture.

To address the knowledge gap, we employ a high-resolution, coupled physical-biogeochemical model to translate global climate and circulation patterns to the estuarine and coastal scale (Figure S1). Using this model, we examine the future evolution of a eutrophic estuary-shelf system up to the year 2100 and quantify the effectiveness of nutrient input reduction and oyster farming in alleviating hypoxia under climate change conditions. Instead of focusing on well-studied hypoxic systems in developed countries (e.g., Baltic Sea, Northern Gulf of Mexico, and Chesapeake Bay), we investigate an emerging seasonal hypoxic system: the Pearl River Estuary and its adjacent shelf off Southern China (referred to as PRE; Figure 1F). The PRE, nourished by China’s second-largest river, the Pearl River, is surrounded by one of the world’s fastest-growing urban areas and supports a prominent oyster aquaculture industry. This makes it a representative coastal system that faces the dual pressures of human disturbance and climate change. Unraveling the future trends of hypoxia in the PRE under different management scenarios can provide valuable insights into various underrepresented coastal systems in developing nations. Additionally, assessing the role of oyster farming in mitigating hypoxia can also reveal the potential of shellfish aquaculture as a sustainable solution to meet food demands while supporting ecosystem restoration.

## 2 Materials and Methods

### 2.1 Observations

We assembled and analyzed long-term monitoring data and multiyear field survey data to assess the biophysical conditions of our study region, create model forcing files, and validate model simulations. The primary data sets include: 1) freshwater discharge of the Pearl River from 1986 to 2021, provided by the Ministry of Water Resources of China (http://xxfb.hydroinfo.gov.cn/); 2) nitrate and phosphate concentrations near the major river outlet, HuMen, from 1991 to 2014, compiled from the literature (Figure S2); 3) concentrations of inorganic nitrogen and phosphorus monitored biweekly near the HuMen outlet from 2014 to 2023; 4) monthly concentrations of total nitrogen and phosphorus near the HuMen outlet from 2015 to 2023, collected by the Department of Ecology and Environment of Guangdong Province (https://gdee.gd.gov.cn/); and 5) 13 summer cruise surveys (June to September) from 2014 to 2021, covering the entire PRE and the adjacent shelf^26,27^ (Figure S3). These summer surveys include key ecosystem variables, such as temperature, salinity, chlorophyll, nitrate, ammonium, phosphate, and dissolved oxygen concentrations.

### 2.2 Physical-Biogeochemical Model of the Coupled Estuary-Shelf System

The three-dimensional (3D) physical-biogeochemical model is based on the Regional Ocean Modeling System (ROMS),^28^ a free-surface, hydrostatic, primitive-equation ocean model. Configured to encompass the Pearl River Estuary and its adjacent shelf in the northern South China Sea^29^ (Figure S1A), the model adopts an adaptive horizontal resolution that gradually decreases from approximately 0.1 km in the estuary and inner shelf to about 1 km at the southern open boundary over the shelf. Additionally, it features 30 terrain-following vertical layers using the S-coordinate system, with refined resolution (<0.2 m) near the surface and bottom. The model utilizes the Mellor-Yamada level 2.5 closure scheme for vertical turbulent mixing, an upwind-biased third-order scheme for horizontal momentum advection, and a third-order accurate, nonoscillatory scheme for tracer advection (HSIMT).^30^

The biogeochemical component (see Figure S1B) is adapted from the expanded N-based model of Fennel et al.,^31^ which includes the cycling of nitrogen, phosphorus, and oxygen. The model consists of 11 state variables: nitrate, ammonium, phosphate, oxygen, chlorophyll, phytoplankton, zooplankton, small detritus, large detritus, and river-delivered terrestrial particulate and dissolved organic matter (POM_terr_ and DOM_terr_). Key processes parametrized in the model include: 1) phytoplankton growth dependent on temperature, light, and nutrient, with ammonium inhibiting nitrate uptake and colimitation effect of nitrogen and phosphorus;^32^ 2) phytoplankton acclimation to changing light intensity by calculating a variable ratio between phytoplankton biomass and chlorophyll; 3) zooplankton grazing based on Holling’s type III (S-shaped) functional response; 4) linear rates for phytoplankton mortality, zooplankton basal metabolism, and remineralization of detritus and terrestrial organic matter; 5) a second-order zooplankton mortality; 6) aggregation of phytoplankton and small detritus to form large detritus; 7) sinking of phytoplankton, detritus, and POM_terr_; 8) light-dependent nitrification; and 9) oxygen production or consumption linked to the aforementioned processes. A mass-conserving and computationally efficient sediment component is implemented following Fennel et al.^31^ and Yu et al.^33^ It assumes that all oceanic sinking organic matter and a fraction of the POM_terr_ reaching the sediment–water interface are instantly remineralized, returning a flux of dissolved constituents to the bottom water. This sediment treatment is similarly applied to organic nitrogen and phosphorus, with a fraction of the deposited organic nitrogen lost to nitrogen gas (N_2_) through denitrification.

The biogeochemical model has been expanded to include the filter-feeding activities of oysters (Figure S1B).^16^ The key processes parametrized in the model related to oysters include: 1) oyster filtration of particulate organic matter (including phytoplankton, zooplankton, small and large detritus, and POM_terr_) based on ambient water salinity, temperature, and oxygen concentration; 2) basal metabolism dependent on temperature, and active respiration associated with food acquisition and assimilation; 3) excretion to ammonium and phosphate; 4) biodeposition from the uningested and/or unassimilated portions of filtered food (i.e., pseudofeces and feces) and oyster mortality; 5) vertical sinking of oyster-derived detritus; 6) remineralization of oyster detritus in both the water column and sediment; and 7) oxygen consumption associated with oyster basal metabolism and remineralization of oyster detritus. The complete set of equations, parameter values, and validation details can be found in Yu and Gan.^16^

The high-resolution coupled estuary-shelf model is one-way nested within the China Sea Multiscale Ocean Modeling System (CMOMS)^34^ (Figure S1C). CMOMS is also based on ROMS and is coupled to the same biogeochemical component as described above. The model domain covers the China Seas (including the South China Sea, East China Sea, Bohai Sea, and Yellow Sea) and the adjacent northwest Pacific Ocean. It has a horizontal resolution of approximately 7 to 10 km and includes 30 terrain-following vertical layers. Details on the CMOMS model setup and validation can be found in Gan et al.^34^ To provide open boundary conditions for the estuary-shelf model, we conducted a CMOMS model projection from 2015 to 2100, utilizing atmospheric fluxes downscaled from the CMIP6 global climate models under the high-emission SSP5-8.5 scenario (see the detailed CMIP6 forcing information in Section 2.3).

For the estuary-shelf model, river discharge rates from 2015 to 2021 were based on historical discharge data, while those from 2022 to 2100 were computed from a Soil and Water Assessment Tool (SWAT) model covering the Pearl River Basin^35^ under the climate forcing of the SSP5-8.5 scenario. River nutrient concentrations are less predictable because they are profoundly affected by environmental policies. Therefore, instead of projecting river nutrient concentrations from watershed models, we based our projections on historical data and hypothesized nutrient management scenarios detailed in Section 2.4.

### 2.3 CMIP6 Forcing

We generated 86-year (2015 to 2100) monthly atmospheric forcing, oceanic boundary variables, and river discharge from the Sixth Phase of the Coupled Model Intercomparison Project (CMIP6) climate model projections under both the moderate-emission SSP2-4.5 scenario and the highest-emission SSP5-8.5 scenario.^36^ The climate models were selected from the list used in the Atlas of the IPCC Sixth Assessment Report (AR6).^37^ Model data were obtained from the CMIP6 database (https://esgf-node.llnl.gov/projects/cmip6/). Atmospheric forcings—including air temperature, air pressure, relative humidity, zonal and meridional wind speed at the surface, rainfall rate, solar shortwave radiation, and cloud fraction—were interpolated onto the CMOMS model grid and then averaged across all available models to create an ensemble mean (Table S1). Similar data processing steps were applied to oceanic velocity, temperature, and salinity to establish boundary forcing for the CMOMS model. In contrast to other atmospheric forcings, the zonal mean atmospheric pCO_2_ projection was derived from long-term greenhouse gas projections from the reduced-complexity climate-carbon-cycle model MAGICC7.0 ^38^ (data available at https://greenhousegases.science.unimelb.edu.au) and then interpolated onto the CMOMS model grid. The ensemble mean river discharge was used for the large-scale CMOMS model only, while the high-resolution estuary-shelf model was forced with river discharge estimates from the previously mentioned SWAT model.

In this study, we present results solely from the SSP5-8.5 experiments. Comparisons of the atmospheric forcing between the two SSP scenarios indicate only a slight increase in domain-averaged air temperature under SSP5-8.5, with minimal difference in wind speed over the China Seas (Figure S4). This allows us to confidently focus our analysis on the SSP5-8.5 scenario.

### 2.4 Model Experiments

The nested ocean modeling system builds upon our previously validated framework,^16,33^ with additional performance metrics presented in Figure S5. The model effectively captures characteristic summer conditions (Figure S5), including the convergence of the southwestward nutrient-rich buoyant jet and the northeastward wind-driven shelf current in the coastal zone. It also reliably represents the stratification that facilitates phytoplankton production (with a surface chlorophyll correlation of 0.38), organic matter accumulation, and hypoxia development (with a bottom oxygen correlation of 0.62). Quantitative analysis of cruise data demonstrates the model’s ability to reproduce key variables with low bias, and the root mean squared error (RMSE) values fall within the range of observational variability. Statistically significant correlations (*p* < 0.05) are found across all variables, ranging from 0.38 to 0.93, with the highest correlations observed for surface nitrate (0.93) and phosphate (0.89). While highly variable estuarine dynamics inherently limit model’s accuracy for patchily distributed variables like chlorophyll, these metrics collectively affirm the model’s utility for analyzing hypoxia mechanisms and making future projections.

To project near-term (2016–2045) and long-term (2071–2100) oxygen dynamics, we employed a computationally efficient “time slice” approach with our validated modeling system. This method simulates discrete seasonal intervals (May to September annually) rather than continuous multidecadal runs, effectively capturing critical summer dynamics when hypoxia typically develops. Each yearly simulation begins in May, initialized from physically consistent states derived from the continuously running CMOMS model, which provides initial and boundary conditions to ensure interannual continuity. This strategy reduces computational demands, preserves interannual variability during the biologically active season, and avoids cumulative numerical round-off errors associated with continuous multidecadal simulations. The method’s reliability has been confirmed by validating model simulations from 2016 to 2024 against the aforementioned cruise observations (Figure S5).

Projections for river nutrient concentrations were based on historical data from 1986 to 2024. Nitrate and phosphate concentrations significantly increased (*p* < 0.05) from 1986 to 2015, followed by a decline after the State Council of China issued the Action Plan for Water Pollution Prevention and Control in 2015,^39^ hereafter referred to as the Nutrient Reduction Scheme 2015 (see Figure S2B). Inspired by these trends, we explored two nutrient management scenarios: the “DecreasingN” scenario assumes robust management that facilitates a persistent decline in river nutrient concentrations until 2100, following the observed decline rate from 2016 to 2023 after the implementation of the Nutrient Reduction Scheme 2015; the “ConstantN” scenario maintains the river nutrient concentrations at 2015 levels until 2100, reflecting a more moderate management approach that curbs the observed increasing trend from 1986 to 2015.

Specifically, in the DecreasingN scenario, we projected river organic and dissolved inorganic nitrogen concentrations to follow the historical declining trend observed from 2016 to 2023 under stringent nutrient reduction strategies. River organic phosphorus concentrations were assumed to follow the same trend as that of organic nitrogen, maintaining a constant stoichiometric ratio in river organic matter. Meanwhile, phosphate concentrations were set to decline in line with the observed trend from 2016 to 2023, leveling off at 1 mmol/m^3^ from 2024 onward, which is approximately the targeted water quality level in mainland China. In the ConstantN scenario, the river nutrient concentrations were maintained at the elevated levels observed in 2015 until 2100. Climatological seasonal cycles of river nutrient concentrations were derived from the biweekly monitoring data collected from 2014 to 2023, and these seasonal patterns were applied to the corresponding nutrient species for all future years.

To evaluate the effectiveness of oyster aquaculture in mitigating hypoxia under climate change, we conducted an “Oyster case” scenario based on the DecreasingN framework. This simulation incorporated a 100 km^2^ oyster cultivation area in the upper water column of the western coastal zone at a typical commercial-scale density of 100 oysters/m^2^. This configuration was selected based on systematic sensitivity experiments by Yu and Gan,^16^ which identified 100 km^2^ as the optimal scale that balances hypoxia mitigation efficiency with ecological carrying capacity in the studied region. The chosen area represents a feasible expansion, equivalent to approximately one-third of Guangdong Province’s current oyster farming area (301 km^2^ in 2022, according to the China Fishery Statistical Yearbook^40^) and a small fraction of China’s total 2349 km^2^ cultivation area. The density of 100 oysters/m^2^ reflects actual regional aquaculture practices, falling within the documented commercial range of 100–350 oysters/m^2^ observed in the adjacent bay.^41^ For our summer-focused simulations, we maintained a fixed oyster biomass of 1 g of dry weight (representing medium-sized oysters) and did not consider the harvest of oysters. While debate exists about whether shellfish biodeposition counteracts the benefits of nutrient removal,^42^ Yu and Gan^16^ showed that this effect is negligible in the coastal zone off PRE. This is attributed to strong wind-driven currents in the area and the significantly reduced deposition flux to the sediment via shellfish filtration, a reduction that substantially lowers sediment oxygen consumption (the dominant oxygen sink in bottom waters of this region^43^).

## 3 Results and Discussion

### 3.1 Climate Change Diminishes Nutrient Reduction Efficacy in Hypoxia Mitigation

As anticipated, the model projects an increase in water-column stratification in the PRE, indicated by a significant (*p* < 0.05) rise in buoyancy frequency (N^2^) (Figure 2A). This increasing stratification is primarily driven by future warming and enhanced freshwater discharge (Figure S2A). Ecosystem responses to these substantial changes in physical conditions are markedly shaped by nutrient management strategies.

**Figure 2:**
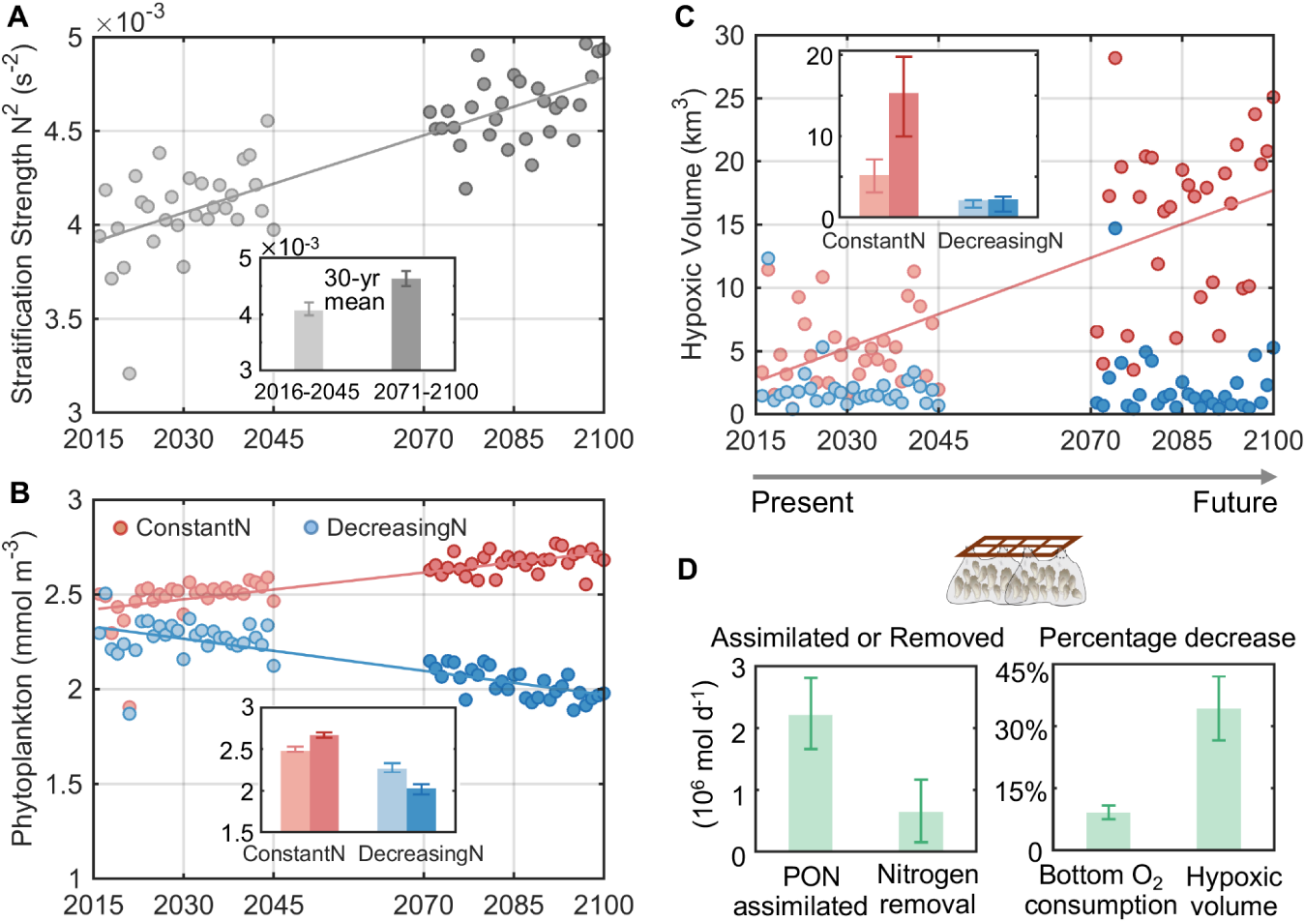
Future projections for different nutrient management scenarios and the impacts of oyster farming. (A–C) Future trends of regionally averaged (A) stratification strength and (B) surface phytoplankton concentration, and (C) regionwide hypoxic volume under various nutrient management scenarios. Each dot represents the summer mean (June to September) for the corresponding year. The inset bar graphs display the 30-year averages for each variable across different scenarios, with the periods 2016 to 2045 shown in lighter color and 2071 to 2100 in darker color. Error bars represent the 25th and 75th percentiles of the data range for each 30-year period. (D) The 60-year average (from 2016 to 2045 and 2071 to 2100) of oyster-assimilated particulate organic nitrogen (PON), nitrogen removed by oysters, and the percentage decrease in bottom oxygen consumption and hypoxic volume induced by oyster farming were relative to the DecreasingN case. Error bars indicate the 25th and 75th percentiles of the data range over the 60 years. Relative changes are calculated exclusively for the western hypoxic center, where oyster farms are located.

In the DecreasingN case, which reflects an ambitious yet realistic nutrient reduction strategy based on 2016–2023 policy achievements (Section 2.4), the steady decline in river nutrient load (Figure S2C) leads to reduced coastal nutrient levels (Figure S6A,B) and decreased phytoplankton biomass (Figure 2B). This results in a 17% decline in bottom water oxygen consumption (Figure S7). However, climate change undermines these gains under SSP5-8.5, as a weakened physical oxygen supply (22% decrease in vertical diffusion; Figure S7) due to increased stratification and decreased solubility outweighs biogeochemical improvements. Consequently, hypoxic volume off the PRE persists, increasing by 6% during 2071–2100 compared to 2016–2045 (Figure 2C), remaining widespread in coastal zones and expanding downstream (Figure S8A,B). While further land-based reductions could theoretically improve oxygen conditions, these marginal gains would require increasingly expensive controls on both point and nonpoint sources, such as advanced wastewater treatment and agricultural runoff mitigation.

Nevertheless, the value of nutrient reduction is clearly demonstrated when compared to the ConstantN case. In this scenario, rising freshwater discharge drives a steady increase in nutrient influx (Figure S2), significantly (*p* < 0.05) fueling phytoplankton biomass growth (Figure 2B) and leading to a 17% rise in bottom water oxygen consumption (Figure S7). Consequently, the ConstantN case predicts a dramatic 195% expansion of hypoxic volume from the earlier 30-year period (2016–2045) to the latter period (2071–2100), far exceeding the 6% increase under DecreasingN. This indicates that higher anthropogenic nutrient input will greatly amplify the effects of climate change on coastal hypoxia. Additionally, hypoxic volume in ConstantN has increased by 147% and 586%, respectively, during the two 30-year periods compared to the DecreasingN case (Figures 2C and S8C,D). This dramatic expansion illustrates that while the DecreasingN scenario cannot fully counteract climate impacts, it remains essential for preventing more catastrophic oxygen depletion.

### 3.2 Effective Hypoxia Mitigation via Oyster Aquaculture

Building on the DecreasingN scenario, we find that adding oyster aquaculture (100 km^2^ farming area at 100 oysters/m^2^) to the western coastal zone (the Oyster case) markedly enhances hypoxia mitigation. The model projects that these oysters assimilate particulate organic nitrogen (PON) at a mean daily rate of (2.2 ± 0.6) × 10^6^ mol/d through filter-feeding. After accounting for nitrogen released via oyster mortality and metabolism, the net nitrogen removal is (0.64 ± 0.57) × 10^6^ mol/d (Figure 2D). This corresponds to an annual oyster-induced N removal rate of 32.9 ± 29.4 g/m^2^/yr, which falls within the observed range of 12 to 152 g/m^2^/yr reported by Rose et al.^44^ and 15.0 to 61.2 g/m^2^/yr in Barrett et al.^45^ By reducing PON in the western coastal zone (Figure 3A,B), oyster farming decreases bottom water oxygen consumption by 10% and shrinks hypoxic volume by 34% compared to the DecreasingN scenario (Figures 2D and S8E,F). These promising results demonstrate that oyster aquaculture can effectively complement land-based efforts to alleviate coastal hypoxia.

**Figure 3:**
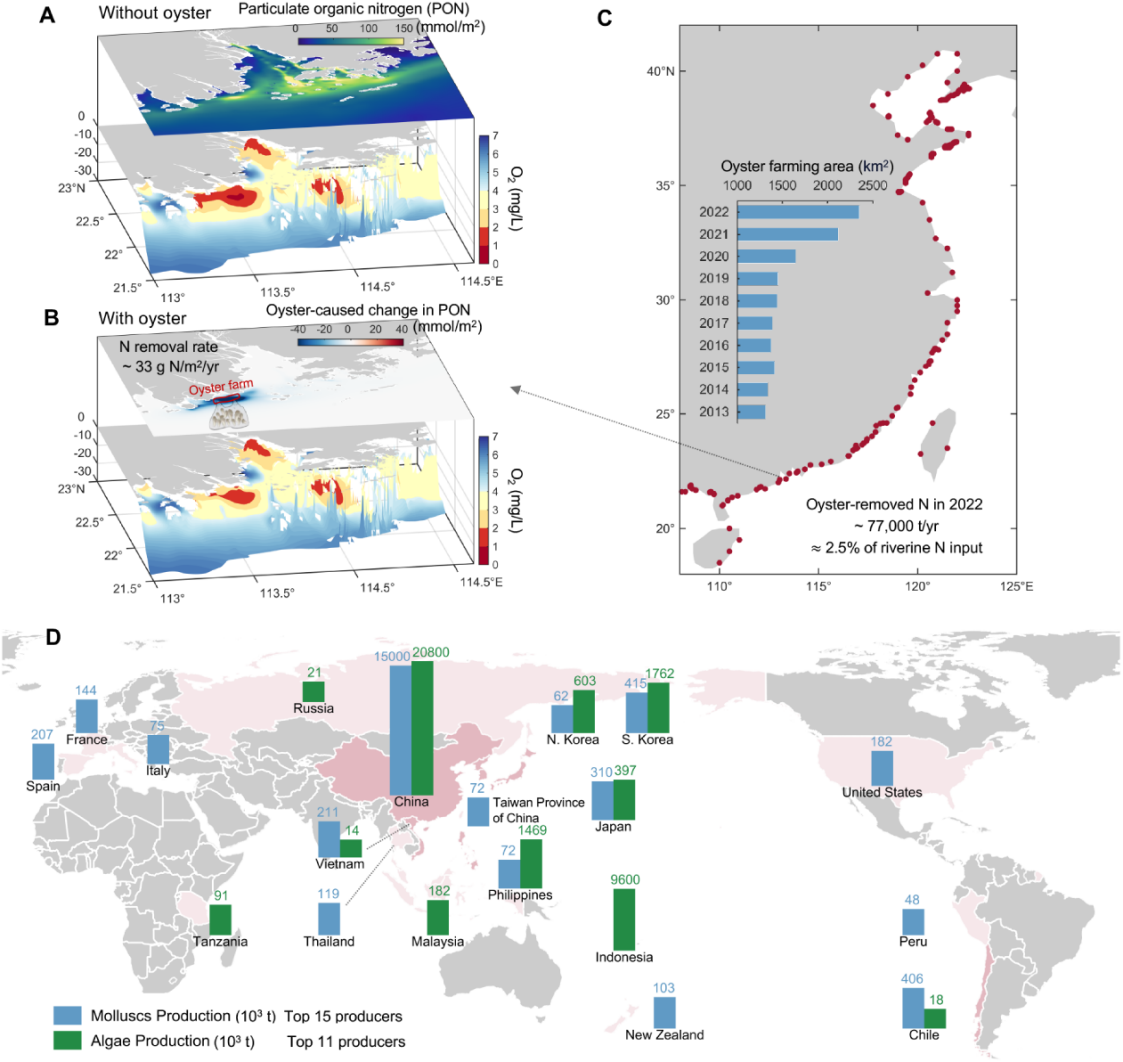
Oyster farming-induced changes, oyster farming sites in China, and major global producers of mollusks and algae. (A) A three-dimensional view of model estimates averaged over 60 years (from 2016 to 2045 and 2071 to 2100), illustrating vertically integrated particulate organic nitrogen concentrations at the top and bottom-water dissolved oxygen concentrations superimposed on the topography below. (B) Similar to A but displaying the oyster farming-induced changes in particulate organic nitrogen at the top. The 100 km^2^ oyster farming area is delineated by the red rectangle. (C) Major oyster farming sites in China, represented by red dots, along with oyster farming areas from 2013 to 2022 shown as blue bars.^40^ (D) Mollusks and algae production by country or region.^20^ Only the top 15 producers of mollusks and the top 11 producers of algae are included. Countries or regions that are the largest producers of both mollusks and algae are colored darker pink.

### 3.3 Global Implications: Nonfed Aquaculture to Complement Land-Based Measures

Despite numerous efforts to reduce nutrient inputs, signs of ecosystem recovery remain rare.^4,6,46^ Climate change further complicates mitigation by impairing oxygen replenishment—a trend evident in the PRE, where increased stratification offsets oxygenation gains from nutrient reductions. This highlights the urgency of adopting complementary strategies, such as nonfed aquaculture (e.g., seaweed and shellfish), to combat deoxygenation.^12,46^ Such a need extends beyond well-known coastal hypoxic zones in developed regions (e.g., the northern Gulf of Mexico,^47^ Baltic Sea,^48^ and Chesapeake Bay^10^) to many underrepresented systems in developing countries experiencing rapidly rising nutrient loads.

Our assessment demonstrates the effectiveness of shellfish aquaculture as a complementary strategy to land-based nutrient reduction for addressing coastal hypoxia under future climate change, based on high-resolution projections. Several modeling considerations merit discussion. While our model captures regional-scale biogeochemical interactions, the fixed oyster biomass assumption for summer simulations does not account for growth-dependent filtration variability or seasonal size dynamics. Physiological rates, including filtration efficiency, naturally increase as the oysters mature. Future model enhancements could incorporate individual-based approaches,^49,50^ particularly for local-scale management where growth dynamics are more influential. For regional-scale, long-term hypoxia projections such as ours, the model strikes a necessary balance between resolution and computational feasibility. The calculated net N removal rates (32.9 ± 29.4 g/m^2^/yr) align with empirical observations (12–152 g/m^2^/yr), validating the model for ecosystem-scale analysis. When scaled to China’s oyster farming area (2349 km^2^ in 2022),^40^ these conservative estimates suggest substantial ecosystem benefits, with a net N removal of ∼7.7 × 10^4^ t/yr, equivalent to 2.5% of the annual riverine N input from China’s eight major rivers (3.1 × 10^6^ t/yr).^51^ At a nitrogen price of 32.3 USD/kg-N (based on European and North American markets),^45^ this removal translates to ∼2.5 billion USD annually.

These projections, while simplified at the organism level, demonstrate meaningful improvements in water quality from shellfish aquaculture. Actual values may vary with site-specific conditions, but the fundamental benefit pattern remains robust. The economic and ecological potential is poised to grow further, given the rapid expansion of oyster farming (Figure 3C) and additional unquantified benefits such as food production,^52^ habitat provision,^45^ shoreline erosion protection,^53^ and carbon sequestration.^54^ These cobenefits warrant further quantitative assessment, establishing shellfish aquaculture as a sustainable development tool that aligns economic and ecological goals in coastal zones.

There is ample potential and incentive for expanding nonfed aquaculture worldwide.^20,52,55^ However, global production of mollusks (e.g., oysters, clams, mussels, and scallops) and algae remains concentrated in a few countries: China produces 83% of mollusks and 59% of algae, with the remainder primarily from South Korea, Chile, Japan, and Vietnam for mollusks and Indonesia, South Korea, the Philippines, and North Korea for algae (Figure 3D). Many developing countries, such as Indonesia, Bangladesh, Uruguay, and Guinea, have substantial capacity for shellfish aquaculture but currently lack large-scale production.^56^ Furthermore, while coastal waters in 132 countries are estimated to have suitable nutrient levels and temperatures for seaweed aquaculture, only 37 are currently engaged in this practice.^54^

The global disparity between the vast potential for nonfed aquaculture and its current concentration in limited regions stems partly from insufficient quantitative assessments of its interactions with coastal ecosystems and dual economic-ecological benefits. While previous studies have mapped the potential of marine aquaculture and highlighted promising opportunities,^54,56^ process-based evaluations of large-scale aquaculture incorporating dynamic coastal processes remain limited. Our coast-resolved PRE framework provides a transferable approach to quantify the benefits of shellfish aquaculture (e.g., nutrient removal and hypoxia mitigation) in diverse coastal systems. Future implementations could extend this approach to include size-dependent physiology of nonfed species beyond oysters (e.g., mussels, clams), carbonate system dynamics for assessing acidification resilience, and sediment biogeochemistry for evaluating benthic-pelagic coupling. These advancements would enable more robust quantification of climate change interactions (e.g., warming and acidification impacts on shellfish growth^57^) and whole-ecosystem effects. Most importantly, integrating these biogeochemical models with socioeconomic frameworks would reveal the full ecosystem service value of nonfed aquaculture, providing policymakers with actionable insights to balance seafood security with marine ecosystem protection against escalating deoxygenation threats.

In summary, we provide a quantitative analysis of the long-term evolution of coastal hypoxia (2016 to 2100) under various nutrient management scenarios, integrating field measurements with a coast-resolved physical-biogeochemical modeling system in an emerging coastal hypoxic zone. Our findings indicate that future climate change can diminish the efficacy of nutrient source control in remediating coastal hypoxia. By demonstrating the effectiveness of oyster aquaculture in combating eutrophication-driven hypoxia, we advocate for incorporating nonfed aquaculture as a complementary strategy alongside conventional land-based measures. This approach aligns well with the global trend of expanding nonfed aquaculture and is particularly beneficial for developing countries seeking sustainable economic growth and ecosystem development.

## Supplementary Material



## Data Availability

The CMOMS model data are available at https://odmp.ust.hk/cmoms/. The CMIP6 data are available at https://aims2.llnl.gov/search. Data used to create figures in the main text and the Supporting Information are available at https://github.com/yuliuqian/Hypoxia-mitigation-efficacy.git. The model used in this study is a community model, ROMS, with codes available at https://www.myroms.org/. The codes modified for this study are available upon request to the corresponding author J.G.

## Notes

Model domain and schematic framework, river forcing, field survey data, future projections of temperature and wind speed, model-data comparisons, future projections of nutrients and hypoxic area, oxygen budget analysis, future projections of hypoxia frequency and oxygen changes, and climate model variables selection (PDF)
