# Supplementary material for: Shellfish Aquaculture Complements Nutrient Reduction in Mitigating Climate-Exacerbated Coastal Hypoxia
Source: Environ Sci Technol. 2025 Aug 21;59(35):18687–95. doi: 10.1021/acs.est.5c06682 (PMC12424163; doi:10.1021/acs.est.5c06682)
Supplement: Supplementary file 1 [file es5c06682_si_001.pdf]

Supporting Information for

Shellfish aquaculture complements nutrient reduction in mitigating climate-exacerbated coastal hypoxia

Liuqian Yu [a,b], Jianping Gan [a,c*], Dou Li [c,d], Weicong Cheng [a,c], Ying Zhang [a,c], Hiusuet Kung[a,c], Chiwing Hui [a,c], and Zheng Chen[b]

[a] Center for Ocean Research in Hong Kong and Macau, The Hong Kong University of Science and Technology, Hong Kong 999077, China

[b] Earth, Ocean and Atmospheric Sciences Thrust, The Hong Kong University of Science and Technology (Guangzhou), Guangzhou 511453, China

[c] Department of Ocean Science and Department of Mathematics, The Hong Kong University of Science and Technology, Hong Kong 999077, China

[d] Department of Earth and Environment, Boston University, Boston, Massachusetts 02215, United States

* Email: magan@ust.hk

This Supporting Information file includes:

Total pages: 11

Figures: 8 (Figures S1 to S8)

Table: 1 (Table S1)

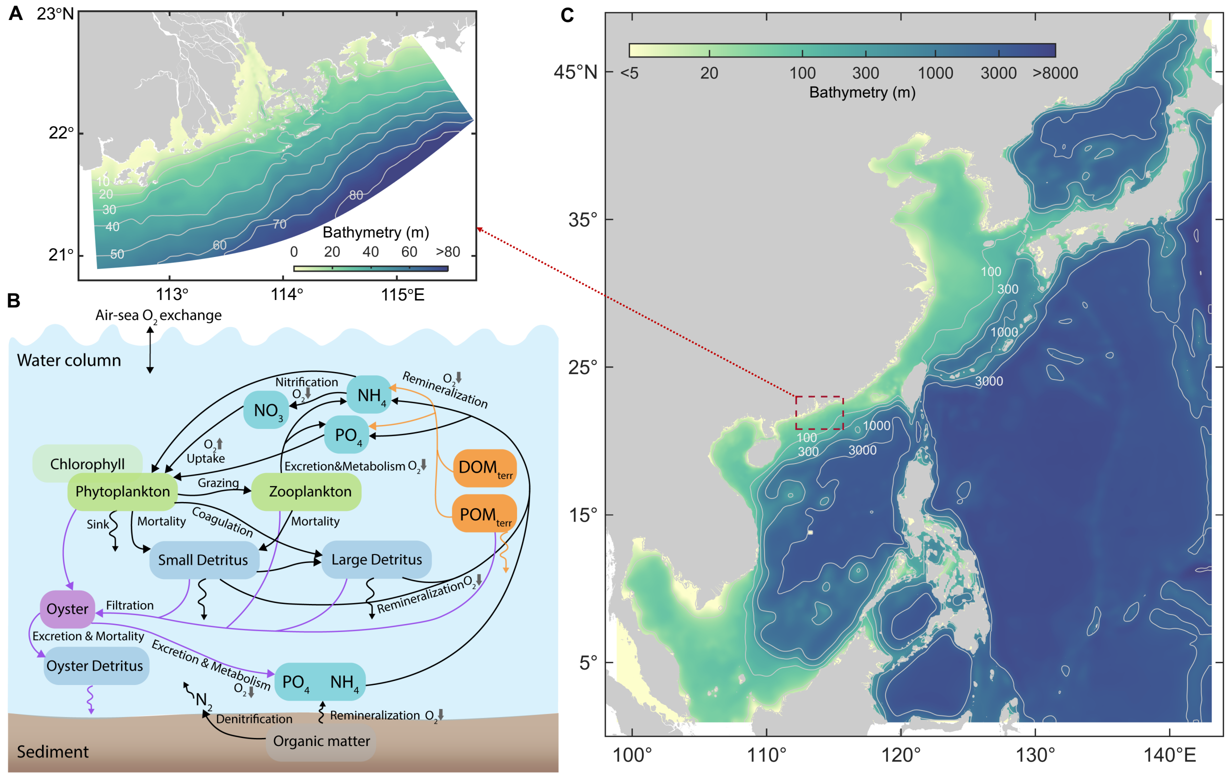

Figure S1. Model domain and schematic framework. (A) High-resolution domain covering the Pearl River Estuary and adjacent shelf, nested within (C) the broader CMOMS (China Sea Multi-scale Ocean Modeling System). Gray contours mark bathymetry (depths in meters). (B) Schematic of the biogeochemical model showing ecosystem state variables (colored rectangles) and major processes (in arrows).

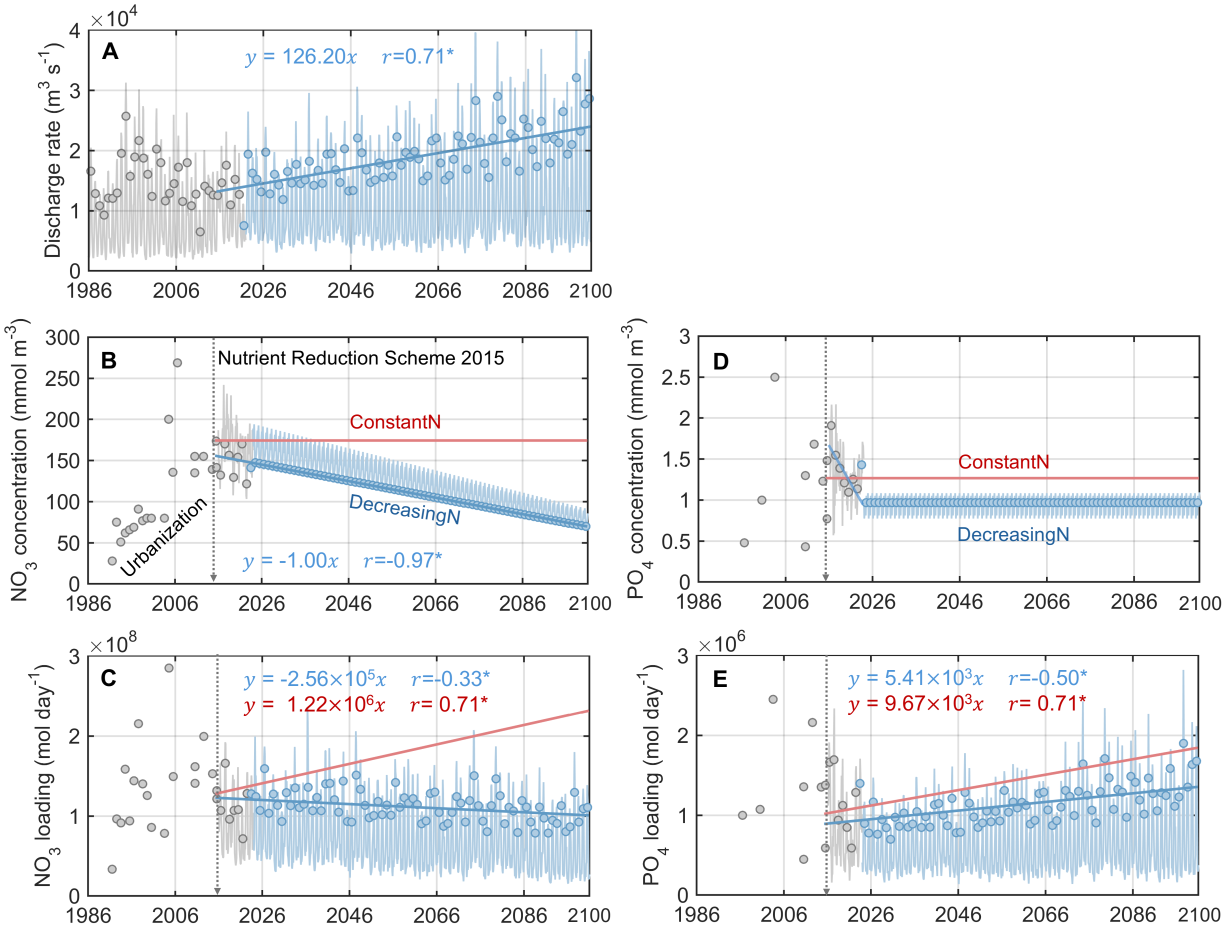

Figure S2. Temporal evolution and trends of river input. (A to C) Temporal evolution and trends of (A) river discharge rate, (B) nitrate (NO_3) concentration, (C) nitrate loading, (D) phosphate concentration, and (E) phosphate loading. Observed data are gray, and data from future projections are blue or red. Thin, solid lines represent monthly variations and dots represent the summer mean (June to September). Also shown in each panel is the linear fit of the summer mean with * denoting the correlation coefficients that are significant at $p<0.05$.

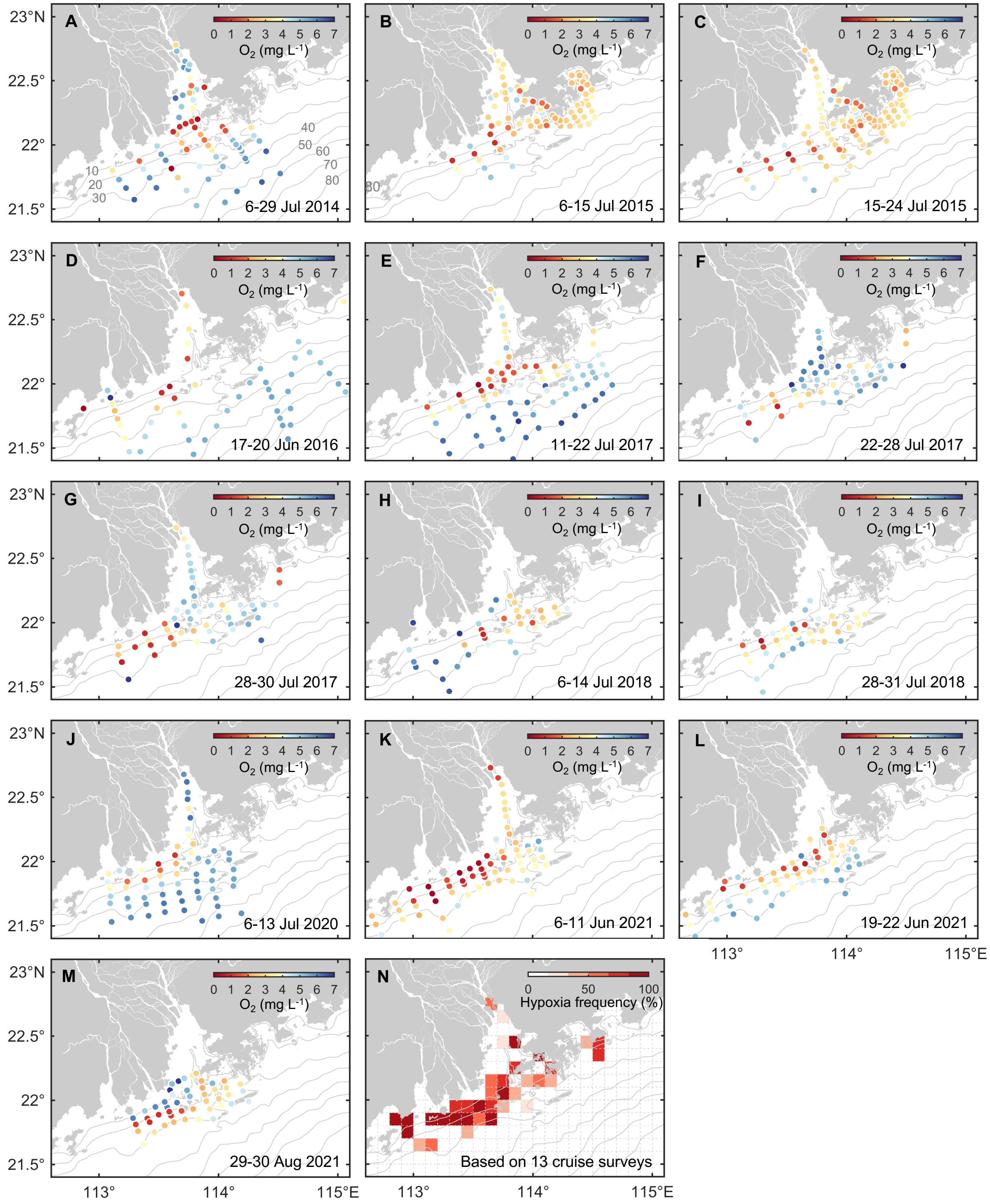

Figure S3. Bottom dissolved oxygen concentration and hypoxia frequency from 13 summer field surveys. Each panel, except for (N), shows the bottom dissolved oxygen concentration from one summer cruise survey, with the dates of the survey indicated at the bottom right corner. Panel

(N) shows the frequency of hypoxia estimated from the 13 surveys. In each panel, gray contours mark bathymetry (depths in meters).

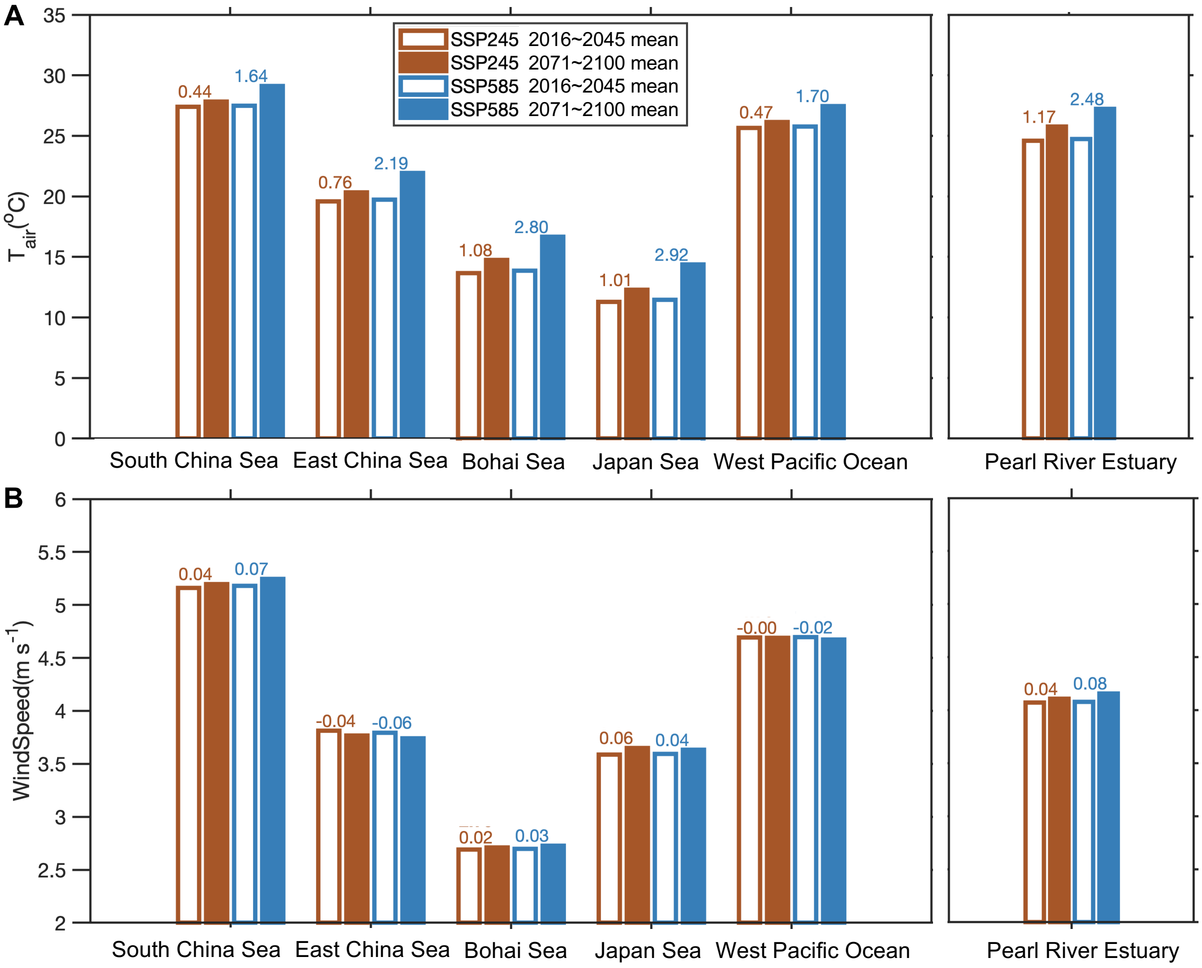

Figure S4. Projected air temperature (A) and wind speed (B) from different Shared Socio-economic Pathways (SSPs) scenarios. Multi-model ensemble mean results from SSP2-4.5 (orange) and SSP5-8.5 (blue) scenarios are spatially averaged over different subregions, including the South China Sea, East China Sea, Bohai Sea, Japan Sea, West Pacific Ocean, and the Pearl River Estuary. The projections are averaged over the near-term (2016-2045; empty bars) and long-term (2071-2100; solid bars) future, respectively. The numbers on the top indicate the difference between long-term and near-term projections.

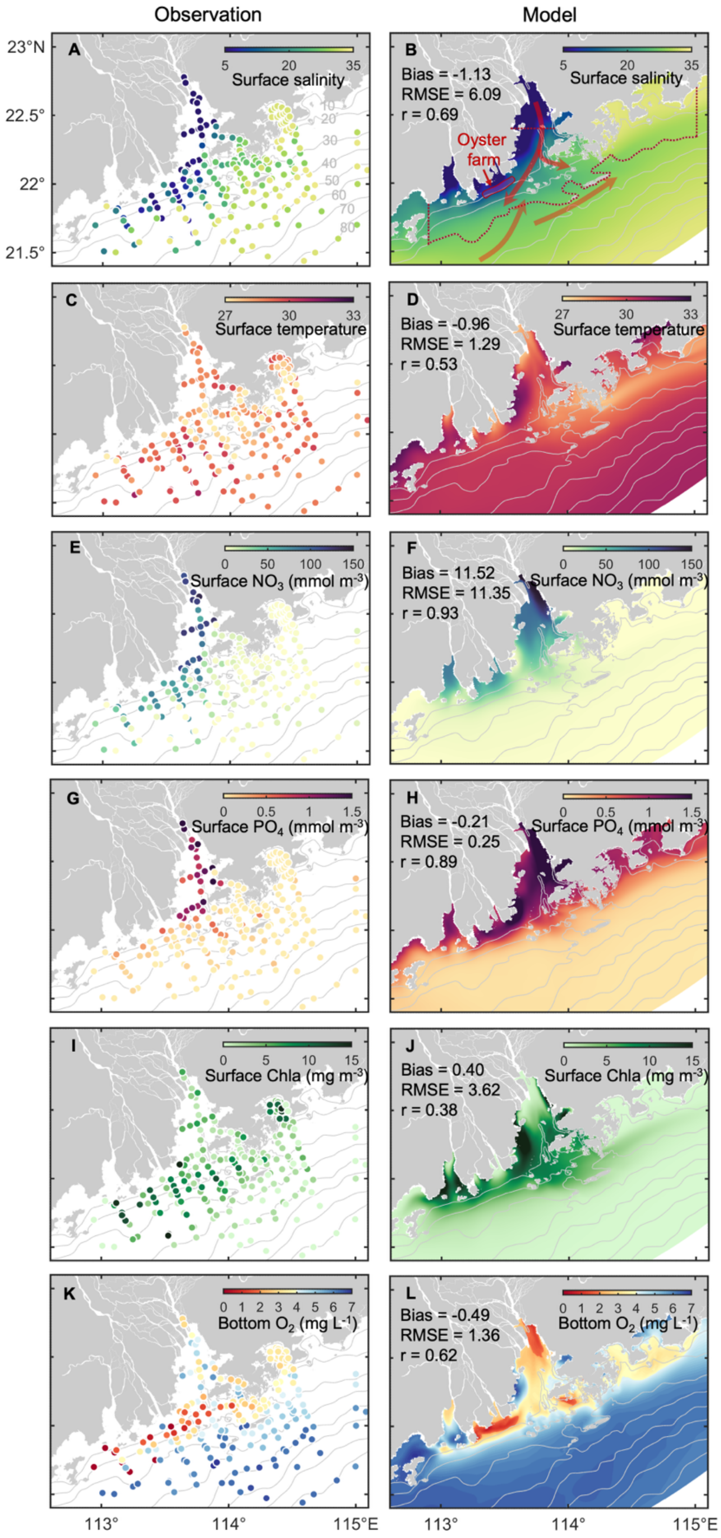

Figure S5. Model-data comparisons of summer surface and bottom water properties. Observed distributions (A, C, E, G, I, K) and model-simulated results (B, D, F, H, J, L) are shown for surface salinity, temperature, nitrate (NO_3), phosphate (PO_4), chlorophyll-a (Chla), and bottom

dissolved oxygen (O_2). Field observations were collected during summer surveys in 2015 and 2017, while model outputs represent summer (June to September) averages from 2016 to 2024. Gray contours mark bathymetric depths. Panel (B) annotates the southwestward buoyant jet and northeastward wind-driven shelf current (red arrows), the coastal zone used for spatial averaging (dashed lines), and the 100 km^2 oyster farm. Quantitative model performance metrics (bias, root mean squared error [RMSE], and correlation coefficient [*r*]) are presented in the right column panels; all reported correlations are statistically significant (p<0.05).

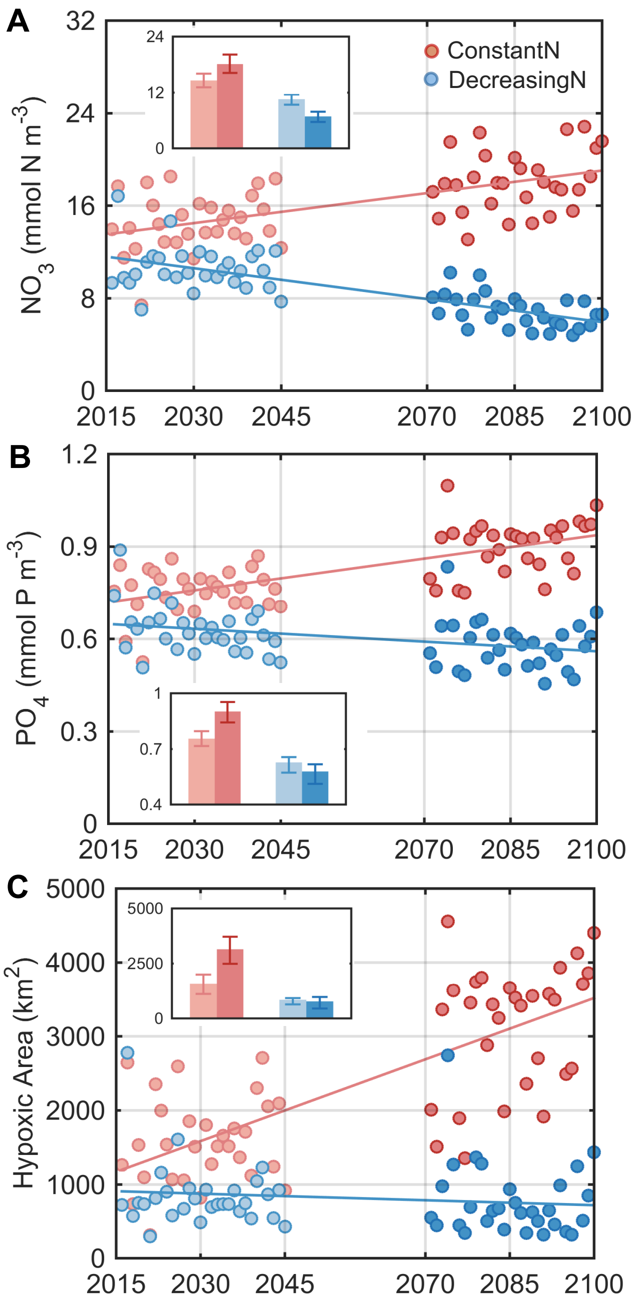

Figure S6. Future projections for different nutrient management scenarios. Future trends of regional averaged surface (A) nitrate (NO_3) concentration, (B) phosphate (PO_4) concentration, and (C) regionwide hypoxic area for different nutrient management scenarios. Inset bar graphs show the 30-year average over the 2016 to 2045 period (lighter color) and the 2071 to 2100 period (darker color) for the respective variable in different scenarios.

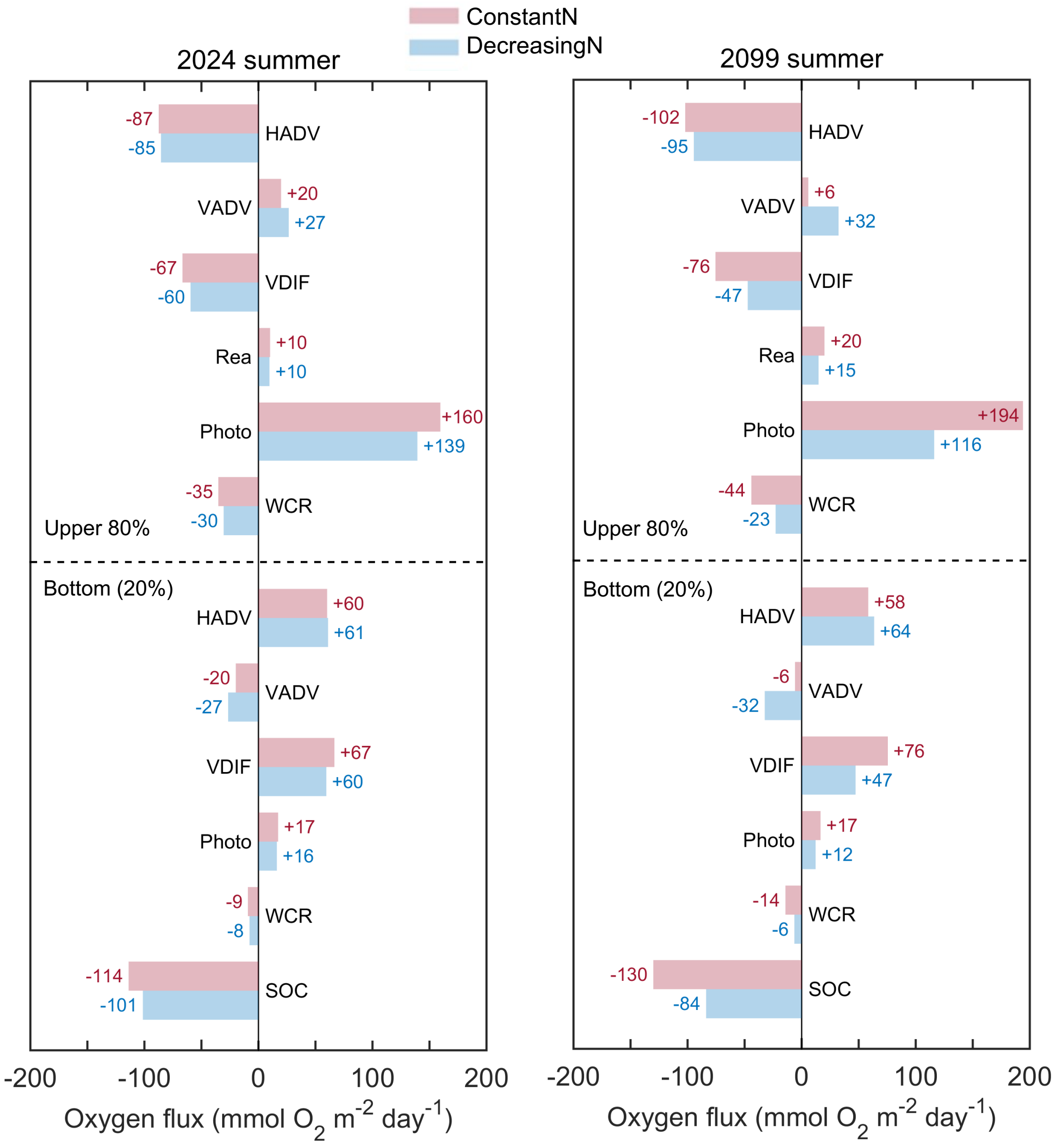

Figure S7. Oxygen budgets in the coastal zone for different scenarios and years. The budget is calculated for the upper 80% and bottom 20% of the water column for different scenarios during the summer months (June to September) of 2024 and 2099, respectively. The coastal zone for spatial average is outlined in Figure S5B.

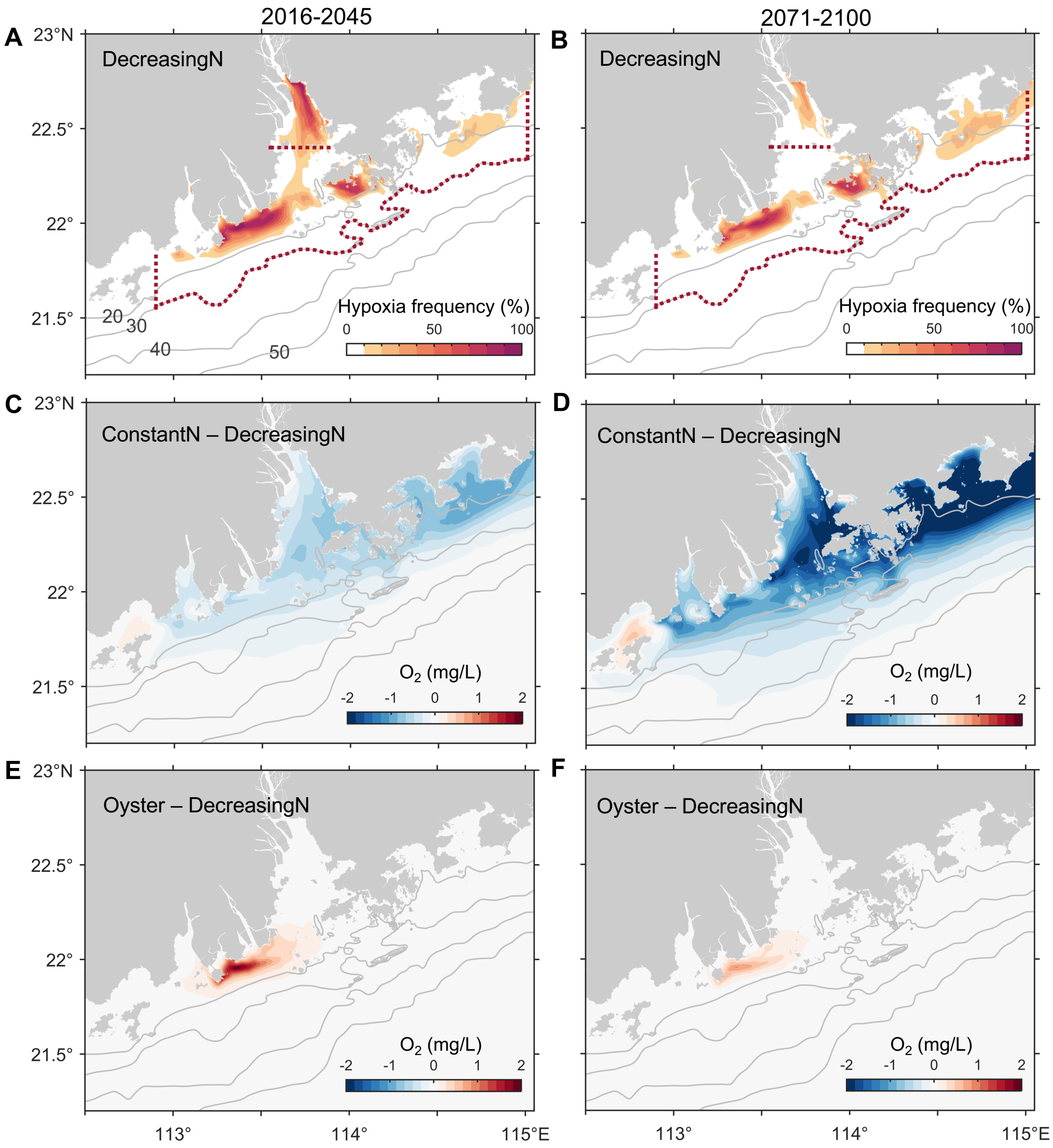

Figure S8. Future projections for hypoxia frequency and difference in bottom oxygen induced by nutrient management scenarios. (A, B) Projected frequency of hypoxia during summer months (June to September) of 2016 to 2045 period (A) and 2071 to 2100 period (B) for the DecreasingN case. Frequency is calculated at each model grid cell by computing the percentage of days with bottom dissolved oxygen < 2 mg/L during the summer months of each 30 years (2016 to 2045 and 2071 to 2100, respectively). The dashed lines outline the coastal zone for the spatial average. (C to F) The deviation from the DecreasingN case of the summer averaged lower-layer oxygen concentration of the (C, D) ConstantN and (E, F) Oyster cases for the 2016 to 2045 period and 2071 to 2100 period, respectively. Here, the lower layer is defined as the bottom 20% of the water column. In each panel, the gray contours mark the bathymetric depths.

Table S1. Variables available in climate models to generate the ensemble mean atmospheric forcing, oceanic boundary conditions, and river discharge for the CMOMS (China Sea Multi-scale Ocean Modeling System) simulations. tas: air temperature; psl: air pressure; hurs: relative humidity; uas: zonal wind speed at the surface; vas: meridional wind speed at the surface; pr: rainfall rate; rsds: solar shortwave radiation; clt: cloud fraction; uo: west-east component of oceanic velocity; uo: west-east component of oceanic velocity; vo: south-north component of oceanic velocity; thetao: temperature; so: salinity; and mrro: total runoff.

MODEL	tas	psl	hurs	uas	vas	pr	rsds	clt	uo	vo	thetao	so	mrro
TaiESM1	☑	☑				☑	☑	☑	☑	☑	☑	☑	☑
AWI-CM-1-1-MR	☑	☑	☑	☑	☑	☑	☑	☑	☑	☑	☑	☑	
BCC-CSM2-MR	☑	☑		☑	☑	☑	☑	☑	☑	☑	☑	☑	☑
CAMS-CSM1-0	☑	☑	☑	☑	☑	☑	☑	☑	☑	☑	☑	☑	☑
FGOALS-g3	☑	☑	☑			☑	☑	☑	☑	☑	☑	☑	☑
CanESM5	☑	☑	☑	☑	☑	☑	☑	☑	☑	☑	☑	☑	☑
IITM-ESM	☑	☑	☑	☑	☑	☑	☑	☑					
CMCC-CM2-SR5	☑	☑	☑	☑	☑	☑	☑	☑	☑	☑	☑	☑	☑
CNRM-CM6-1	☑	☑	☑	☑	☑	☑	☑	☑	☑	☑	☑	☑	☑
CNRM-CM6-1-HR	☑	☑	☑	☑	☑	☑	☑	☑	☑	☑	☑	☑	☑
CNRM-ESM2-1	☑	☑	☑	☑	☑	☑	☑	☑	☑	☑	☑	☑	☑
ACCESS-ESM1-5	☑	☑	☑	☑	☑	☑	☑	☑	☑	☑	☑	☑	☑
ACCESS-CM2	☑	☑	☑	☑	☑	☑	☑	☑	☑	☑	☑	☑	☑
EC-Earth3	☑	☑	☑	☑	☑	☑	☑	☑	☑	☑	☑	☑	☑
EC-Earth3-Veg	☑	☑	☑	☑	☑	☑	☑	☑	☑	☑	☑	☑	☑
EC-Earth3-Veg-LR	☑	☑	☑	☑	☑	☑	☑	☑	☑	☑	☑	☑	☑
INM-CM4-8	☑	☑	☑	☑	☑	☑	☑	☑	☑	☑	☑	☑	☑
INM-CM5-0	☑	☑	☑	☑	☑	☑	☑	☑	☑	☑	☑	☑	☑
IPSL-CM6A-LR	☑	☑	☑	☑	☑	☑	☑	☑	☑	☑	☑	☑	☑
KIOST-ESM	☑	☑	☑	☑	☑	☑	☑	☑	☑	☑	☑	☑	☑
MIROC-ES2L	☑	☑	☑	☑	☑	☑	☑	☑	☑	☑	☑	☑	☑
MIROC6	☑	☑	☑	☑	☑	☑	☑	☑	☑	☑	☑	☑	☑
HadGEM3-GC31-LL	☑	☑	☑	☑	☑	☑	☑	☑	☑	☑	☑	☑	☑
UKESM1-0-LL	☑	☑	☑	☑	☑	☑	☑	☑	☑	☑	☑	☑	☑
MPI-ESM1-2-LR	☑	☑	☑	☑	☑	☑	☑	☑	☑	☑	☑	☑	☑
MRI-ESM2-0	☑	☑	☑	☑	☑	☑	☑	☑	☑	☑	☑	☑	☑
CESM2	☑	☑	☑			☑	☑	☑	☑	☑	☑	☑	☑
CESM2-WACCM	☑	☑	☑			☑	☑	☑	☑	☑	☑	☑	☑
NorESM2-LM	☑	☑	☑			☑	☑	☑	☑	☑	☑	☑	☑
NorESM2-MM	☑	☑	☑			☑	☑	☑	☑	☑	☑	☑	☑
KACE-1-0-G	☑	☑	☑	☑	☑	☑	☑	☑					☑
GFDL-ESM4	☑	☑	☑	☑	☑	☑	☑	☑	☑	☑	☑	☑	☑
GFDL-ESM4	☑	☑	☑	☑	☑	☑	☑	☑			☑	☑	☑
NESM3	☑	☑		☑	☑	☑	☑	☑	☑	☑	☑	☑	
